# Bilateral facial nerve palsy as a presentation of coexisting neuroborreliosis and post-acute COVID-19 syndrome

**DOI:** 10.3325/cmj.2023.64.440

**Published:** 2023-12

**Authors:** Katarina Blažina, Ivan Martinez, Manuela Foro Znika

**Affiliations:** 1 Department of Neurology, University Hospital Center Zagreb, School of Medicine, University of Zagreb, Zagreb, Croatia; 2 Special Hospital for Medical Rehabilitation Krapinske Toplice, Krapinske Toplice, Croatia

## Abstract

Bilateral simultaneous facial nerve palsy is an extremely rare condition that may be induced by infection (bacterial, viral, or fungal) or noninfective causes (autoimmune, traumatic, or structural). The treatment depends on the underlying disorder, and, if it is introduced on time, the disease is in most cases completely reversible. We report on a patient with bilateral simultaneous facial nerve palsy without an obvious cause. The possible causes were SARS-CoV-2 infection and postvaccination syndrome. After we excluded the SARS-CoV-2 infection, a wide range of diagnostic tests were conducted. Magnetic resonance imaging after gadolinium intravenous application showed bilateral facial nerve enhancement. Serological tests revealed *Borrelia burgdorferi*, and the result was confirmed by an enzyme-linked immunosorbent assay (IgM positivity). After 14 days of antibiotic therapy, the symptoms resolved completely without sequelae. This report shows that the symptoms of bilateral simultaneous facial nerve palsy may completely resolve if thorough clinical investigation and an appropriate early treatment are applied.

Bilateral simultaneous facial nerve palsy (BS-FNP) is a very rare condition with an incidence of 1 per 5 000 000 people. It accounts for fewer than 2% of all FNP cases (1,2). BS-FNP is characterized by the paralysis of both sides of the face muscles within a month. Its etiology differs from that of FNP: while unilateral FNP is mostly idiopathic, BS-FNP is usually symptomatic and caused by various potentially treatable conditions (cytomegalovirus, Epstein-Barr virus, varicella- zoster virus, herpes simplex 1 and 2, HIV, and SARS-CoV-2; various bacterial causes of meningitis like *Leptospira, Borrelia burgorferi, Klebsiella pneumoniae*, and *Toxoplasma gondii*) (2,3). The causes may also be non-infectious conditions such as Guillain-Barré syndrome (GBS), leukemia, sarcoidosis, cranial base fracture, intracranial neoplasms, idiopathic cranial neuropathy, Melkerson-Rosenthal syndrome, demyelination diseases, Kawasaki disease, and post-vaccination syndrome (2-4). To the best of our knowledge, this is the first described case of coexisting neuroborreliosis and post-acute COVID-19 syndrome.

## Case report

We report on a patient with BS-FNP and bilateral enhancement of the facial nerve on MRI after gadolinium contrast injection, proven coexisting post-acute COVID-19 syndrome, and neuroborreliosis.

**Figure 1:**
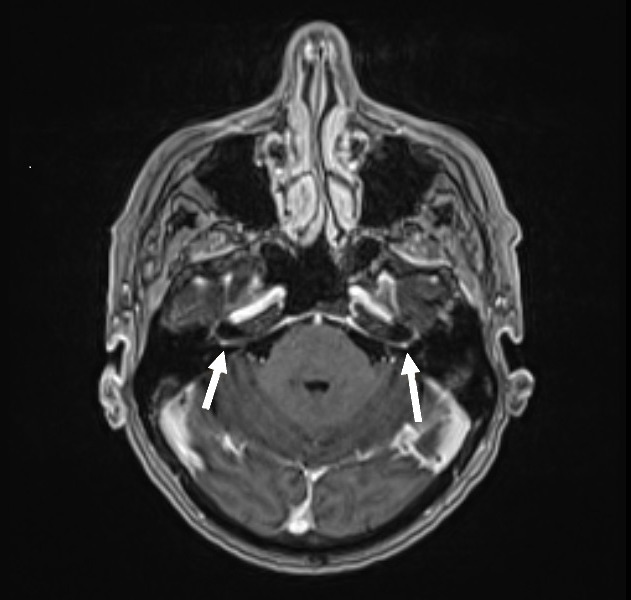
Magnetic resonance imaging (1.5T) findings: an axial contrast-enhanced thin section (after i.v. gadolinium application). The T1-weighted image shows bilateral intrameatal facial nerve enhancement (arrows).

**Figure 2:**
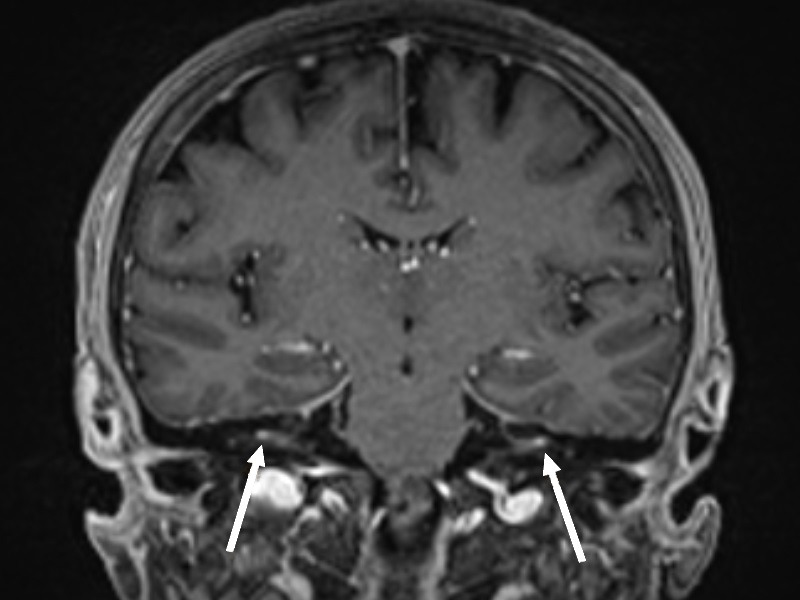
Magnetic resonance imaging (1.5T) findings: a coronal contrast-enhanced thin section (after i.v. gadolinium application). The T1-weighted image shows bilateral intrameatal facial nerve enhancement (arrows).

On July 23, 2022, a 68-year-old woman was admitted to the emergency department with a sudden onset of bilateral facial muscle weakness, drooling, slurred speech, and sensitivity to loud noises. Her medical history included arterial hypertension controlled by antihypertensive therapy. She, her husband, and her daughter developed COVID-19 in November 2021 during the delta variant predominance. SARS-CoV-2 infection had been confirmed by positive reverse-transcription polymerase chain reaction (PCR). The patient had been vaccinated with three doses of the COVID-19 vaccine tozinameron (BNT162b2). At the physical exam at admission, no trauma, fever, rash, or other infectious or oncological diseases were noted. A neurological examination revealed BS-FNP; other cranial nerves were intact. Her muscle strength and tone on extremities were normal, as were her deep tendon reflexes. No pathological reflexes were induced. There were no signs of cerebellar impairment. Gait and walking were normal. Vital functions at admission were within the reference range. Emergency brain computed tomography (CT) showed no signs of disease. Laboratory tests and extended laboratory investigation (all the tests are shown in Table 1) were unremarkable. At admission, real-time PCR for SARS-CoV-2 was negative. Lumbar puncture revealed pleocytosis (159 lymphocytes/mm^3^) with elevated proteins (0.6 g/L) and normal glucose and lactate levels in the cerebrospinal fluid (CSF). MRI of the brain showed gadolinium enhancement of both facial nerves in the internal auditory canal (Figure 1 and Figure 2). Serological testing (serum and CSF) for neurotrophic viruses excluded the infection with cytomegalovirus, Epstein-Barr virus, *Toxoplasma gondii*, varicella-zoster, herpes simplex 1 and 2, *Klebsiella pneumoniae*, and SARS-CoV-2 viruses. Enzyme-linked immunosorbent assay showed positive IgG antibodies against *Borrelia burgdorferi* in serum and the CSF. Western blot confirmed the presence of *Borrelia burgdorferi* IgM bands. The patient responded well to i.v. ceftriaxone therapy lasting 14 days. She was discharged without neurological sequelae.

**Table 1:** The timeline of diagnostic tests and interventions

Date	Diagnostic tests
November 2, 2021	SARS-CoV-2 infection was confirmed by a positive reverse transcription polymerase chain reaction.
July 23, 2022	The patient was admitted with sudden onset of bilateral facial muscle weakness, drooling, slurred speech, and sensitivity to loud noises. Emergency brain computed tomography (CT) showed no signs of disease. Magnetic resonance imaging of the brain showed gadolinium enhancement of both facial nerves in the internal auditory canal. Laboratory tests, including complete blood cell count, liver enzymes, electrolytes, glucose, C-reactive protein, and coagulation profile were unremarkable. Lumbar puncture showed pleocytosis (159 lymphocytes /mm^3^) with elevated proteins (0.6 g/L) and normal glucose and lactate levels in the cerebrospinal fluid (CSF). Empirical antimicrobal therapy with ceftriaxone was started.
July 24, 2022	Extended laboratory investigation was conducted including serum tumor markers: carbohydrate antigen 19-9, cytokeratin 19, neuron-specific enolase, carcinoembryonic antigen, carbohydrate antigen 125, alpha-fetoprotein; immunological analysis for antinuclear antibody, rheumatoid factor, anti-double-stranded DNA antibodies, and extractable nuclear antigen antibodies.
August 4, 2022	Results of serological testing (serum and CSF) for neurotrophic viruses excluded infections with cytomegalovirus, Epstein-Barr virus, *Toxoplasma gondii*, varicella-zoster, herpes simplex 1 and 2, *Klebsiella pneumoniae*, and SARS-CoV-2 viruses. We found positive IgG antibodies against *Borrelia burgdorferi* in serum and CSF by an enzyme-linked immunosorbent assay. Western Blot found *B.burgdorferi* IgM bands.
August 10, 2022	The patient was discharged without neurological sequelae.

## Discussion

This case report shows neuroborreliosis to be the main etiological factor for BS-FNP with strongly suspected post-acute COVID 19 coexistence. COVID-19 presents with a wide spectrum of neurological symptoms, affecting both the central and peripheral nervous system. Symptoms associated with COVID-19 are divided into three major groups: neurological manifestation in acute infection, post-acute COVID-19 syndrome, and vaccine-induced syndrome (5). FNP has been described as a part of SARS-CoV-2 mRNA vaccine-induced syndrome (5). Only several reports so far have mentioned FNP in the context of post-acute COVID-19 syndrome (6). FNP and BS-FNP in post-acute COVID-19 syndrome can be seen as a part of GBS as a result of inappropriate immune response and polyneuropathy, but in rare cases as a non-GBS symptom (5,6).

After COVID-19 was excluded as a cause, serological tests revealed an infection with *Borrelia burgdorferi*. Lyme disease is a multisystem infectious disease spread by ticks infected by the spirochete *Borrelia burgdorferi*. Neuroborreliosis can have various clinical manifestations and mimic other neurological diseases (both of the central and peripheral nervous system). A common problem in diagnosing Lyme disease is that, at the time of clinical presentation, patients frequently do not recall having had erythema migrans. For example, only 50% of patients with Lyme neuroborreliosis in Europe remembered that they had had erythema migrans, and only 25% showed the rash or its remnants at the time of clinical presentation (7).

Unilateral FNP and BS-FNP can be visualized with MRI as an enhanced area after intravenous gadolinium contrast application, usually in the distal intrameatal nerve segment position, which appears because of blood-brain barrier breakdown due to an inflammatory process (8). The intensity and duration of gadolinium-contrast FN enhancement do not correlate with symptoms severity, and it persists long after complete recovery (8).

In our patient, the diagnosis was established after extended diagnostic procedures. Electromyography and nerve conduction studies of the facial nerves showed bilateral, predominantly demyelinating lesion of all three studied branches (frontal, zygomatic, and mandibular) with prolonged distal latencies and compound motor action potential amplitudes within the reference range. Cerebrospinal fluid analysis showed pleocytosis and positive IgM bands on Western blot.

The question remains whether BS-FNP was induced solely by neuroborreliosis or also by post-acute COVID-19 syndrome. Post-acute COVID-19 syndrome might have triggered neuroborreliosis damage, but BS-FNP may have been co-induced by both entities.

In conclusion, in the case of BS-FNP, a wider diagnostic spectrum should be considered because the disease has many possible causes (infectious and non-infectious). In times such as the COVID-19 pandemic, it is easy to overlook some of the rare causes of BS-FNP or even coexisting entities. We would like to emphasize the importance of a thorough clinical investigation in the cases of BS-FNP because the results can sometimes be surprising. An appropriate early treatment can lead to symptoms resolution without long-lasting neurological sequelae.
